# Structural Characterization of Cis– and Trans–Pt(NH_3_)_2_Cl_2_ Conjugations with Chitosan Nanoparticles

**DOI:** 10.3390/molecules27196264

**Published:** 2022-09-23

**Authors:** Penparapa Chanphai, Gervais Bérubé, Heidar-Ali Tajmir-Riahi

**Affiliations:** Department of Chemistry, Biochemistry and Physics, University of Québec at Trois-Rivières, Trois-Rivières, QC G9A 5H7, Canada

**Keywords:** chitosan, cis and trans–Pt(II), delivery, loading efficacy, thermodynamic analysis

## Abstract

The conjugation of chitosan 15 and 100 KD with anticancer drugs cis– and trans–Pt (NH_3_)_2_Cl_2_ (abbreviated cis–Pt and trans–Pt) were studied at pH 5–6. Using multiple spectroscopic methods and thermodynamic analysis to characterize the nature of drug–chitosan interactions and the potential application of chitosan nanoparticles in drug delivery. Analysis showed that both hydrophobic and hydrophilic contacts are involved in drug–polymer interactions, while chitosan size and charge play a major role in the stability of drug–polymer complexes. The overall binding constants are *K*_ch–15–cis–Pt_ = 1.44 (±0.6) × 10^5^ M^−1^, *K*_ch–100–cis–Pt_ = 1.89 (±0.9) × 10^5^ M^−1^ and *K*_ch–15–trans–Pt_ = 9.84 (±0.5) × 10^4^ M^−1^, and *K*_ch–100–trans–Pt_ = 1.15 (±0.6) × 10^5^ M^−1^. More stable complexes were formed with cis–Pt than with trans–Pt–chitosan adducts, while stronger binding was observed for chitosan 100 in comparison to chitosan 15 KD. This study indicates that polymer chitosan 100 is a stronger drug carrier than chitosan 15 KD in vitro.

## 1. Introduction

**Scheme 1:**
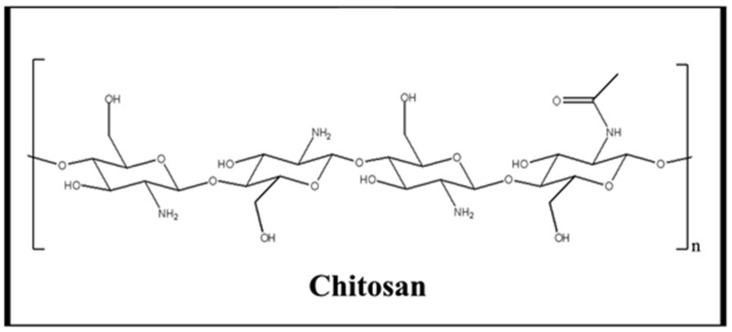
Chemical structure of chitosan.

Biodegradable polymers, such as chitosan, have been extensively studied as carriers for therapeutic protein and gene delivery systems [1,2]. Chitosan (Scheme 1) is a natural polymer obtained by the deacetylation of chitin [3]. It is a non-toxic, biocompatible, and biodegradable polysaccharide. Chitosan nanoparticles have gained increased attention as drug delivery carriers because of their superior stability, low toxicity, and simpler and milder preparation method, thus providing versatile routes for the administration of drugs [4,5]. The deacetylated chitosan backbone of glucosamine units has a high density of charged amine groups, allowing strong electrostatic interactions with proteins and genes that carry an overall negative charge at neutral pH conditions [4,6]. The fast-expanding research regarding the valuable physicochemical and biological properties of chitosan has led to the recognition of this cationic polysaccharide as an important natural polymer for drug delivery [6,7,8,9,10,11,12,13]. Therefore, it was of major interest to study the conjugation of cis– and trans–Pt with chitosan and the potential application of chitosan nanoparticles in the delivery of Pt drugs.

Cisplatin has been widely used for solid tumor chemotherapy but its trans–platin isomer proved to be ineffective due to its instability and rapid deactivation by hydrolysis in the blood circulation [14,15,16,17]. It is commonly believed that the mode of action of this anticancer agent relies mainly on the formation of intrastrand cross-linked DNA adducts that block DNA replication and transcription, finally causing cancer cell death. The use of nanodelivery tools has also grown and many different strategies have been explored to deliver platinum compounds in vitro and in vivo [17,18]. Trans-Pt also causes DNA damage. It is relatively more reactive than cis–Pt and, consequently, it can be deactivated before reaching DNA. Furthermore, its trans geometry does not allow for the formation of closed di–adducts on DNA [19,20]. These facts explain the lower biological activity of trans–Pt in comparison to cis–Pt. The literature shows that trans–Pt forms different types of DNA lesions: 35% of monofunctional adducts, 53% of intrastrand crosslinks (mainly 1,3-GXG and 1,3-AXG where X is any nucleotide), and 12% of interstrand crosslinks [21]. It is also reported that trans–Pt DNA damage can be more easily recognized and repaired than that produced by cis–Pt [22]. It is important to note that there are many platinum antitumor complexes with trans geometry possessing antitumor activity [22]. A comparative study on the interaction of cis–Pt and trans–Pt with DNA and RNA found that aggregation occurs at high concentrations and that DNA remains in the B-family conformation and RNA retains its A-family conformation [23]. It is evident that a substantial body of work was performed to study cis–Pt and trans–Pt interaction with DNA, their mechanism of action, and cell processing of platinated DNA [24,25]. However, little is known about cis–Pt and trans–Pt delivery by natural and synthetic polymers. Hence the rationale for this investigation.

Here, we present a spectroscopic and thermodynamic analysis of the conjugation of cis– and trans–Pt with chitosan 15 and 100 KD in an aqueous solution at pH 5–6, using a constant polymer concentration and various drug contents. Structural information regarding Pt drug binding sites, binding efficacy, and the stability of Pt–chitosan complexes are discussed in this study.

## 2. Results and Discussion

### 2.1. Stability of Drug–Chitosan Complexes by UV Spectroscopy 

**Figure 1:**
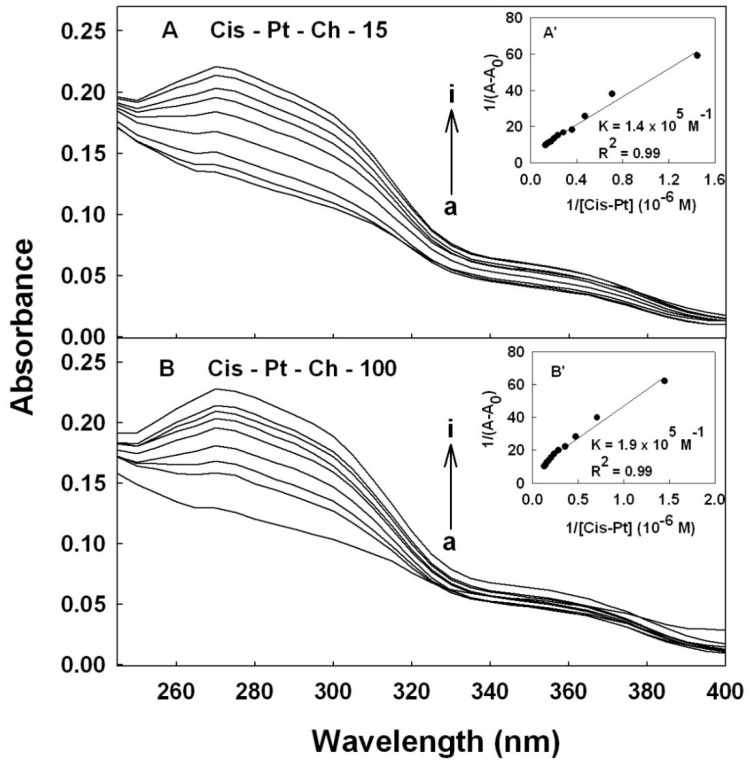
UV–visible spectra of chitosan nanoparticles with cis–Pt with free chitosan at 60 µM (a) and cis–Pt at 1, 5, 10, 20, 30, 40, 60, and 80 µM (b–i). Inset: plot of 1/(A_0_ − A) vs. (1/drug concentration) and binding constant (*K*) for cis–Pt–polymer complexes. (**A**) Cis–Pt–Ch–15, (**B**) Cis–Pt–Ch–100.

**Figure 2:**
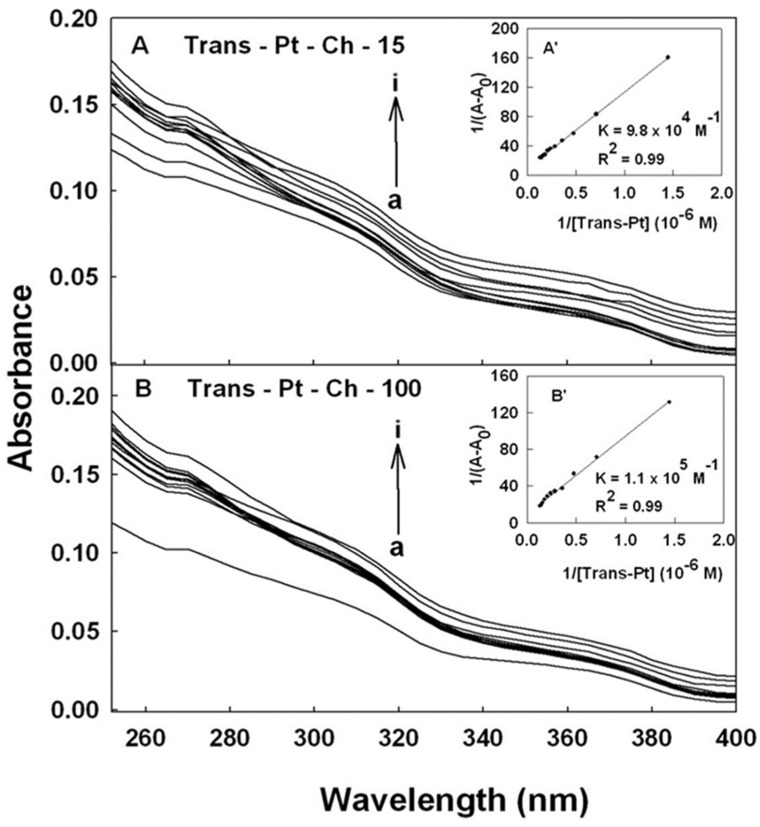
UV–visible spectra of chitosan nanoparticles with trans–Pt with free chitosan at 60 µM (a) and trans–Pt at 1, 5, 10, 20, 30, 40, 60, and 80 µM (b–i). Inset: plot of 1/(A_0_ − A) vs. (1/drug concentration) and binding constant (*K*) for trans–Pt–polymer complexes. (**A**) Trans–Pt–Ch–15, (**B**) Trans–Pt–Ch–100.

The binding constants of cis– and trans–Pt with chitosan nanoparticles were determined as described in Section 3 [26,27]. The increasing drug concentration resulted in a constant decrease in UV light of chitosan at 270 nm (Figure 1). This is in agreement with the aggregation of chitosan upon drug interaction (Figure 1 and Figure 2). The double reciprocal plot of 1/(A_0_ − A) vs. 1/(drug concentration) is linear and the binding constants (*K*) are estimated from the ratio of the intercept to the slope (Figure 1 and Figure 2), where A_0_ is the initial absorbance of the free polymer at 270 nm and A is the recorded absorbance of complexes at different drug concentrations. The binding constants were of Kch–15–cis–Pt = 1.44 (±0.6) × 10^5^ M^−1^, Kch–100–cis–Pt = 1.89 (±0.9) × 10^5^ M^−1^ and Kch–15–trans–Pt = 9.84 (±0.5) × 10^4^ M^−1^, Kch–100–trans–Pt = 1.15 (±0.6) × 10^5^ M^−1^ (Figure 1 and Figure 2 and Table 1). Cis–Pt forms more stable complexes than trans–Pt–chitosan adducts, while stronger binding was observed for chitosan 100 in comparison to chitosan 15 KD. Thermodynamic analysis of drug–chitosan interactions with regard to hydrophobic and hydrophilic contacts are discussed below.

### 2.2. Thermodynamic Analysis of Drug–Chitosan Adducts

**Figure 3:**
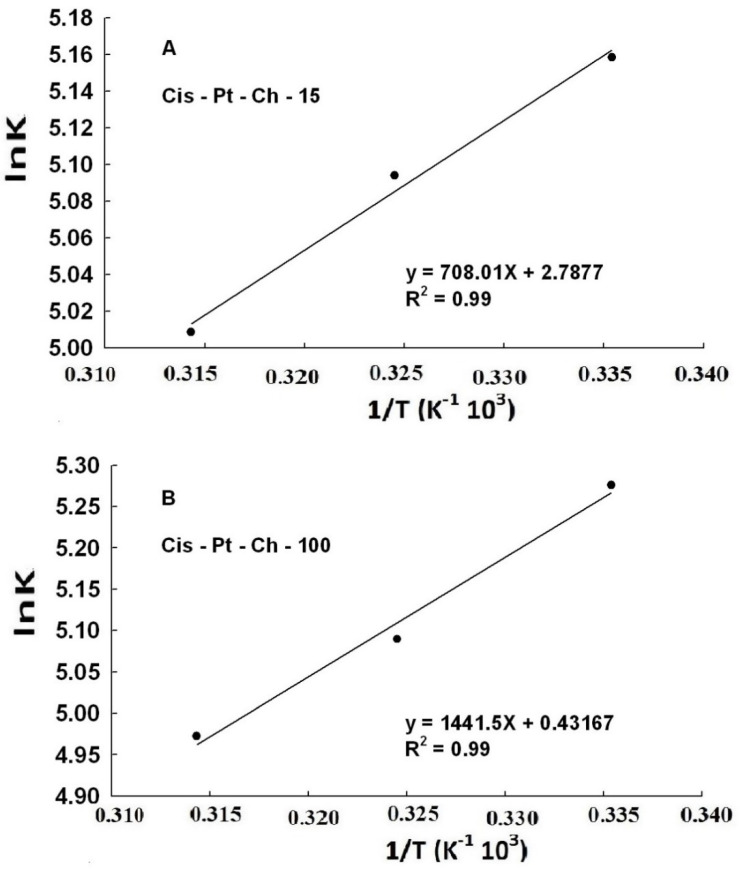
lnK vs. 1/T for chitosan with cis–Pt conjugates: (**A**) Cis–Pt–Ch–15; (**B**) Cis–Pt–Ch–100.

**Figure 4:**
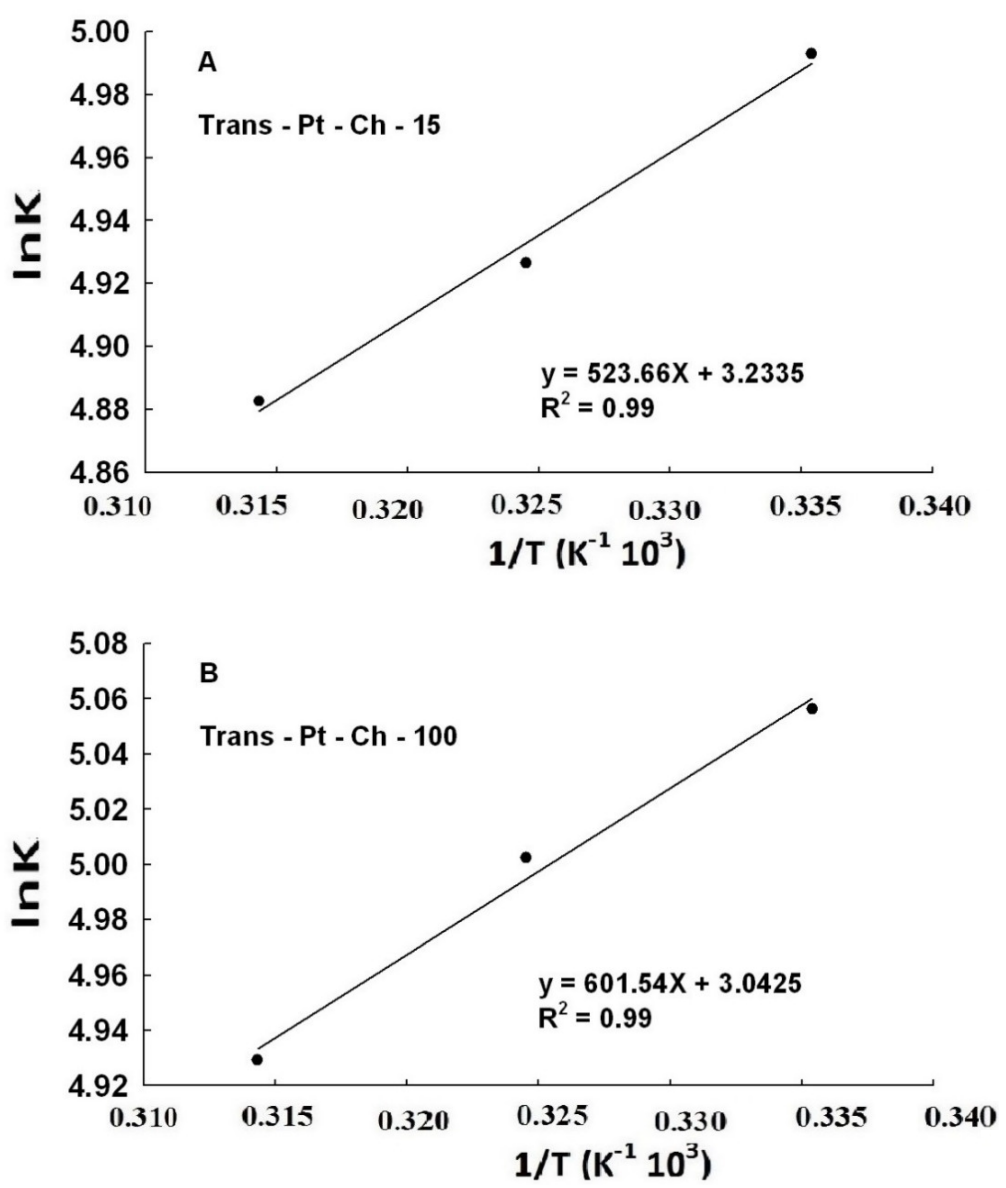
lnK vs. 1/T for chitosan with trans–Pt conjugates: (**A**) Trans–Pt–Ch–15; (**B**) Trans–Pt–Ch–100.

The conjugation of Pt drugs was further analyzed by thermodynamic analysis. Based on the data of Δ*H*^0^ and Δ*S*^0^, the nature of drug–chitosan interactions was determined [28,29,30,31]. The thermodynamic parameters for the interaction of the Pt drugs and chitosan nanoparticles at 298.15, 308.15, and 318.15 *K* are shown in Figure 3 and Figure 4 and Table 2. The negative sign of Δ*G*^0^ showed that the binding process between drug and polymer is spontaneous. The drug–polymer complexes have negative Δ*H*^0^, showing that the complex formation between the polynucleotide and the Pt drugs is an exothermic reaction. The negative Δ*H*^0^ and positive Δ*S*^0^ for drug adducts indicate that ionic interactions are observed in the Pt–chitosan complexation [28,29,30,31]. Therefore, the enthalpy provides more contribution to Δ*G*^0^ than entropy, which indicates that the binding process is enthalpy driven (Table 2). 

The binding efficacy for drug–chitosan conjugates was determined as reported [32]. The binding efficacy was estimated to be 75% for cis–Pt and 65% for trans–polymer complexes.

### 2.3. FTIR Spectra of Pt Drug–Chitosan Complexes 

**Figure 5:**
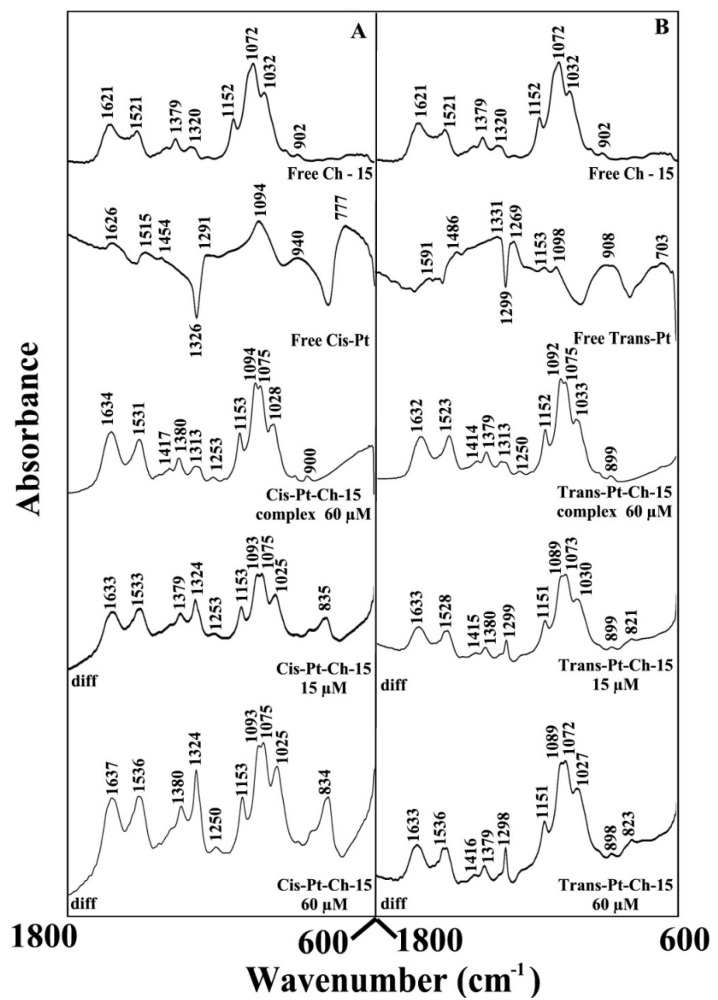
FTIR spectra in the region of 1800–600 cm^−1^ of hydrated films (pH 5–6) for chitosan–15 kD with cis–Pt (**A**) and trans–Pt (**B**) with chitosan (60 µM) and its Pt conjugates with difference spectra (diff.) (bottom two curves) obtained at different drug concentrations (indicated on the figure).

**Figure 6:**
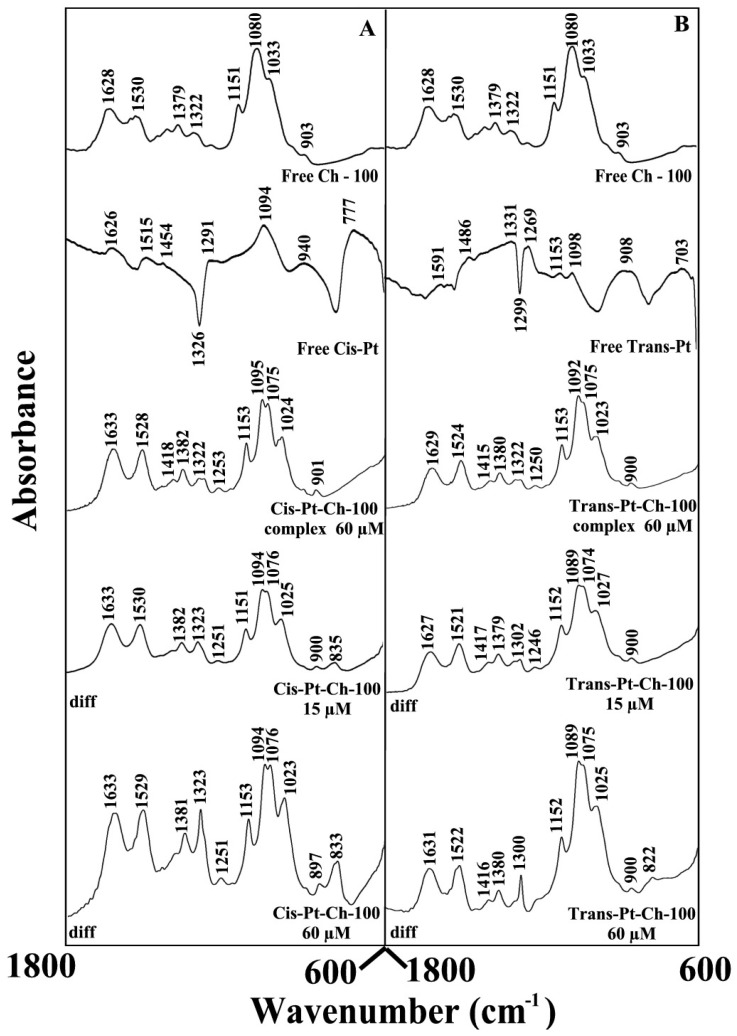
FTIR spectra in the region of 1800–600 cm^−1^ of hydrated films (pH 5–6) for chitosan–100 KD with cis–Pt (**A**) and trans–Pt (**B**) with chitosan (60 µM) and its Pt conjugates with difference spectra (diff.) (bottom two curves) obtained at different drug concentrations (indicated on the figure).

The chitosan interactions with cis– and trans–Pt were characterized by infrared spectroscopy and its derivative methods. The spectral shifting and intensity variations in protein and chitosan amide I band at 1656–1630 cm^−1^ (mainly C=O stretch) and amide II band at 1547–1525 cm^−1^ (C–N stretching coupled with N–H bending modes) [9,33] were monitored upon Pt interaction. The difference spectra ((chitosan solution + Pt drug solution)–(chitosan solution)) were obtained, in order to monitor the intensity variations in these vibrations and the results are shown in Figure 5 and Figure 6.

At a low polymer concentration (15 µM), an increase in intensity was observed for the protein amide I at 1658–1656 and amide II at 1544–1543 cm^−1^ in the difference spectra of the chitosan–cis–Pt and chitosan–trans–Pt complexes (Figure 5A,B and Figure 6A,B, and diff., 15 µM). The positive features located in the difference spectra for the amide I and II bands at 1657, 1546 cm^−1^ (ch–15–Pt) and 1661, 1551 cm^−1^ (ch–100–Pt) are due to the increase in the intensity of chitosan amide I and amide II bands upon Pt drug interaction (Figure 5 and Figure 6 and diffs 15 µM). This increase in the intensity for the polymer amide I and amide II bands is due to chitosan binding to Pt drug via C=O, C–N, and N–H groups (hydrophilic contacts). 

As the chitosan concentration increased to 60 µM, strong positive features were observed for the amide I band at 1656, 1546 (ch–15–Pt) and 1654, 1546 (ch–100–Pt), upon chitosan complexation (Figure 5A,B and Figure 6A,B, and diff., 60 µM). In addition, spectral shifting was observed for chitosan amide I at 1637–1632 and amide II at 1540–1526 cm^−1^ upon polymer–Pt complexation (Figure 5A,B and Figure 6A,B, and 60 µM complexes). The observed spectral shifting and intensity variations in the amide I and amide II bands are due to chitosan binding via C–O, C–N, and NH_2_ groups [9].

## 3. Materials and Methods

### 3.1. Materials 

Purified chitosan 15 and 100 KD (90% deacetylated) were from Polysciences Inc. (Warrington, FL, USA) and used as supplied. Cis– and trans–Pt(NH_3_)Cl_2_ were purchased from Sigma Chemical Company (St. Louis, MO, USA) and used as supplied. Other chemicals were of reagent grade and used without further purification.

#### 3.1.1. Preparation of Cis– and Trans–Pt Adducts with Chitosan Nanoparticles

Chitosan nanoparticles were prepared as reported in an earlier study [34,35]. Chitosan was dissolved in acid solution (40 mg/mL or 0.5 mM) containing 10 mM acetate buffer (pH 5–6). HCl (1 mmol) was used for this preparation. An appropriate amount of cis–Pt or trans–Pt was dissolved in water solution and diluted in Tris–HCl. Pt–chitosan complexes were characterized by UV, FTIR, and thermodynamic analysis.

#### 3.1.2. UV–Visible Spectroscopy

The UV–Vis spectra were recorded on a Cary 60 UV–Visible spectrophotometer with a slit of 2 nm and a scan speed of 400 nm min^−1^. Quartz cuvettes of 1 cm were utilized. The absorbance measurements were performed at pH 7.2 by keeping the concentration of chitosan constant (60 µM), while increasing drug concentrations (1 to 60 µM). The binding constants of drug–chitosan adducts were determined as reported [26].

The drug–chitosan binding constants were calculated according to the following equations:

Chitosan + drug ⇔ Chitosan:drug(1)

*K* = [Chitosan: drug complex]/[chitosan]_unc._ [drug]_unc._ (2)

wherein

drug = cis–Pt or trans–Pt and unc. stands for uncomplexed.

The values of the binding constants *K* were obtained from the chitosan absorption at 270 nm according to the methods published in the literature [26] where the bindings of various ligands to biomolecules were described. For weak binding affinities, the data were treated using linear reciprocal plots based on the following equation:

(3) $$\frac{1}{A-A_{0}}=\frac{1}{A-A_{0}}+\frac{1}{K(A_{\infty }-A_{0})}\cdot \frac{1}{C_{\mathrm{ligand}}}$$

where A_0_ is the absorbance of chitosan at 270 nm in the absence of ligand, A_∞_ is the final absorbance of the ligated-chitosan, and A is the recorded absorbance at different ligand concentrations. The double reciprocal plot of 1/(A − A_0_) vs. 1/C*_ligand_* is linear and the binding constant (*K*) can be estimated from the ratio of the intercept to the slope [26].

#### 3.1.3. FTIR Spectroscopy

Infrared spectra were recorded on a FTIR spectrometer (Impact 420 model), equipped with deuterated triglycine sulfate (DTGS) detector and KBr beam splitter, using AgBr windows. Solutions of drugs were added dropwise to chitosan solution with constant stirring to ensure the formation of a homogeneous solution and with drug concentrations of 15, 30, and 60 µM, and a final chitosan concentration of 60 µM. Spectra were collected after 2 h incubation of polymer with drug solution at room temperature, using hydrated films. Interferograms were accumulated over the spectral range 4000–600 cm^−1^ with a nominal resolution of 2 cm^−1^ and 100 scans. Bands from chitosan in-plane vibrational frequencies were used as standard reference in spectral subtraction [9,33].

## 4. Conclusions

Pt drugs bind chitosan via hydrophilic and hydrophobic contacts with more stable complexes formed for cis–Pt than for trans–Pt. Chitosan 100 forms stronger complexes with Pt than with chitosan 15 KD. As chitosan size becomes larger, protein self–aggregation occurs, which induces a major effect on polymer–drug interactions. From this study, it is established that chitosan 100 is a stronger Pt carrier than chitosan 15 KD. The former could be used to transport Pt–based drugs, potentially enhancing efficacy and drug availability in vivo.

## Figures and Tables

**Table 1:** Variations in the binding constants for Ch–15 and Ch–100 kD with cis–Pt and trans–Pt at different temperatures.

Complexes	Temperature (K)	Binding Constant *K* (mol/L)^−1^
	298.15	1.44 × 10^5^
Cis–Pt–Ch–15	308.15	1.24 × 10^5^
	318.15	1.02 × 10^5^
	298.15	1.89 × 10^5^
Cis–Pt–Ch–100	308.15	1.23 × 10^5^
	318.15	9.39 × 10^4^
	298.15	9.84 × 10^4^
Trans–Pt–Ch–15	308.15	8.44 × 10^4^
	318.15	7.63 × 10^4^
	298.15	1.14 × 10^5^
Trans–Pt–Ch–100	308.15	1.01 × 10^5^
	318.15	8.50 × 10^4^

**Table 2:** Thermodynamic parameters for Ch–15 and Ch–100 kD with cis–Pt and trans–Pt.

	Thermodynamic Parameters			
Complexes	Δ*H*^0^ (kJ. mol^−1^)	Δ*S*^0^ (J. mol^−1^. K^−1^)	TΔ*S*^0^ (kJ. mol^−1^)	Δ*G*^0^ (kJ. mol^−1^)
			6.91	−12.80 (298.15 K)
Cis–Pt–Ch–15	−5.89	23.18	7.14	−13.03 (308.15 K)
			7.37	−13.26 (318.15 K)
			1.07	−13.05 (298.15 K)
Cis–Pt–Ch–100	−11.98	3.59	1.11	−13.09 (308.15 K)
			1.14	−13.13 (318.15 K)
			8.02	−12.37 (298.15 K)
Trans–Pt–Ch–15	−4.35	26.88	8.28	−12.64 (308.15 K)
			8.55	−12.91 (318.15 K)
			7.54	−12.54 (298.15 K)
Trans–Pt–Ch–100	−5.00	25.3	7.79	−12.80 (308.15 K)
			8.05	−13.05 (318.15 K)

## Data Availability

Not applicable.

## Sample Availability

Samples of the compounds are not available from the authors.

## Abbreviations

Ch	chitosan
cis– and trans–Pt	Cis– and trans–Pt(NH_3_)_2_Cl_2_
FTIR	Fourier transform Infrared
